# 4D Printing of Renewable Materials Derived from Glycerol and Maleic Anhydride with Tunable Thermal, Mechanical, and Fluorescent Properties

**DOI:** 10.1021/acsomega.6c01077

**Published:** 2026-04-08

**Authors:** Gabriel I. dos Santos, Caroline Gaglieri, Rafael T. Alarcon, Aniele de Moura, Fernanda B. dos Santos, Gilbert Bannach

**Affiliations:** † Faculdade de Ciências, 28108 Universidade Estadual Paulista (UNESP), Bauru 17033-260, Brazil; ‡ Instituto de Química de São Carlos, 28133 Universidade de São Paulo (USP), São Carlos 13566-590, Brazil; § Centro de Ciências Exatas e de Tecnologia, Universidade Federal de São Carlos (UFSCar), São Carlos 13565-905, Brazil

## Abstract

The demand for sustainable polymers in additive manufacturing has driven the search for renewable precursors capable of enabling advanced functionalities in 3D and 4D printing. In this work, renewable thermosets were developed from an α,β-unsaturated polyester derived from glycerol and maleic anhydride (PPH) combined with 2-hydroxyethyl methacrylate (HEMA), yielding photocurable resins with a theoretical biobased content of 97% and sustainable formulation scores above 43. The resins were processed via vat photopolymerization and subjected to a secondary UV postcuring step to induce tunable properties. The postcuring process increased monomer conversion, significantly enhancing thermal stability, maximum tensile strength, and hardness. Moreover, the materials exhibited luminescent behavior, attributed to polymerization-induced emission (PIE) and aggregation-induced emission (AIE) effects, marking the emergence of nonconjugated luminescent polymers. In addition, the PPH30-based polymer demonstrated high shape memory performance, with shape fixity and recovery ratios above 90% after multiple cycles, confirming its potential for 4D printing applications. Altogether, these findings highlight the feasibility of employing renewable feedstocks to design smart materials with tunable mechanical, thermal, and optical properties. This approach aligns with the sustainable development goals (SDGs), offering new pathways for greener manufacturing of advanced functional materials for engineering, biomedical, and optical applications.

## Graphical abstract

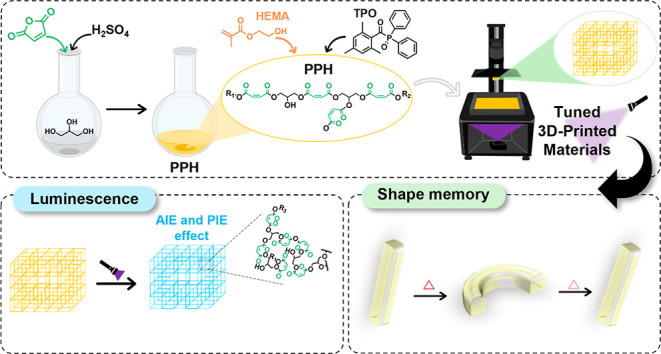

## Introduction

As technology and manufacturing techniques evolve, new ways of producing materials have been developed. One example is additive manufacturing, also known as 3D printing, which is a process able to produce objects in different formats for different applications.^1^ Among the 3D printing techniques, those involving a photopolymerization reaction are called vat photopolymerization 3D printing (VP). Due to the chemical character of VP, it stands out as a promising field for producing rigid or flexible materials with high resolution, as well as in short printing times.^1,2^ Overall, in VP, an object with the desired format is designed in CAD software, which generates a printing file that is sent to the printer. The printing process occurs when a liquid photocurable resin inside the vat is irradiated with light, producing a thin, solid layer multiple times until the entire object is built.^1–3^ Thus, besides the construction of materials with complex shapes, VP is a promising way for producing smart materials.

By definition, smart materials are those that receive, transmit, or process a stimulus and exhibit a response to it.^4^ Common stimuli include strain, stress, temperature, pH, magnetism, pressure, radiation, and so on.^4,5^ Meanwhile, the responses observed can be shape-changing, self-healing, rheological-changing, production of electric charge, and so on.^6^ Within the smart materials classes, stimuli-responsive polymers (SRP) or simply “smart polymers” have been explored for application in several fields, such as biology,^5,7–9^ medicine,^10–13^ and robotics.^14–16^ Besides, when the 3D printing process is used to obtain materials that are capable of producing structural changes (at the macro or molecular level), triggered by external stimuli over time, we have 4D printing.^17,18^ This concept was reported for the first time in 2013, and since then, 4D printing has been widely explored in the development of programmable materials for different fields such as aerospace, automotive, clothing, construction, military defense, and biomedicine.^17^ The process behind 4D printing is based on the use of smart materials in the printing process, which further generates objects that can undergo shape changes over time and then be “programmed” according to specific demands. Due to these features, the 4D printing market is estimated to reach approximately USD 488 million by 2025.^17^

Materials with tunable properties are those that, when exposed to specific stimuli, can acquire new functionalities or enhance existing ones in a permanent manner. The tunable properties may include mechanical, thermal, optical, magnetic, and fluorescent characteristics.^19–23^ The tuning process can be induced through thermal, electrical, magnetic, or photonic treatments, which trigger structural changes that result in altered or improved performance. Owing to these capabilities, tunable materials are highly promising for a wide range of applications, as they can be tailored on demand to be suitable for specific requirements.

As sustainable demands have increased in recent years, the development of polymers by using renewable resources has attracted attention from industry and academia. The development of 4D printing and tunable materials would not differ, as most used monomers come from fossil-based resources, which contribute to white pollution.^24^ In this sense, some researchers have explored the use of renewable resources for producing biobased materials by 4D printing. Constant et al.^25^ explored limonene and myrcene in the development of photocurable resins for 3D printing. In this approach, they homo- and copolymerized the aforementioned compounds and used the resulting oligomers to formulate resins with suitable viscosity and reactivity for photopolymerization. Furthermore, the materials obtained presented suitable capabilities for shape memory behavior, with some polymers presenting a shape fixation greater than 99%. Thus, due to these characteristics, 4D printing could be achieved by using renewable and greener resins. As shown by Brooks et al.^26^ thermosetting materials with shape memory behavior and biobased content between 25 and 65% were 4D-printed from polyester-based resins obtained from limonene oxide, cyclic anhydrides, and a reactive diluent from eugenol. This approach resulted in materials with tunable physical properties and tailorable degradation profiles. In another work, Li et al.^27^ explored itaconic acid and polycaprolactone in the formulation of biobased resins for 4D printing of pH-responsive materials. These approaches not only contribute to the development of material sciences but are also aligned with the United Nations Sustainable Development Goals (SDGs), specifically with Goals 12, “Responsible Consumption and Production,” and 9, “Industry, Innovation and Infrastructure”.^28^

Although renewable resources offer a sustainable pathway for developing greener 4D printing and tunable materials, the exploration of different renewable sources for creating such materials remains limited, with only a few studies reported in the literature. In a previous work,^29^ we synthesized an acrylic polyester derived from glycerol and maleic anhydride (PPH), which was used in the development of renewable and photocurable resins. After a reactivity study, these resins containing different amounts of PPH were applied in vat photopolymerization 3D printing to produce semi-interpenetrating polymer networks (semi-IPNs) with distinct thermal and mechanical properties. Although solid materials were obtained, residual double bonds were still present after the printing and postcuring processes, which resulted in polymers with low thermal stability, maximum tensile strength, and hardness values. Therefore, the objective of this work is to demonstrate the potential of these greener resins for 4D printing by performing a postprinting tuning process with ultraviolet light, enabling the production of materials with new or enhanced properties. This approach resulted in important advances in 4D printing and in the development of renewable tunable materials, since polymers with luminescent behavior, shape memory, and improved thermal and mechanical properties were obtained. Moreover, this work offers potential for achieving SDGs 9 and 12.

## Experimental Section

### Materials

Glycerol (GLY) was purchased from Merck. Maleic anhydride (MA; 99%), sulfuric acid (H_2_SO_4_; 95%), pentaerythritol tetrakis­(3-mercaptopropionate) (PT3M; 95%), diphenyl­(2,4,6-trimethylbenzoyl)­phosphine oxide (TPO; 97%), 2-Hydroxyethyl methacrylate (HEMA; 99%) were purchased from Sigma-Aldrich and used as received.

### Acrylic Polyester Synthesis

The potentially biobased acrylic polyester (PPH) synthesis was conducted according to the optimized conditions reported in the literature.^29^ Glycerol and maleic anhydride were reacted in a 2:3 molar ratio (GLY/MA) in the presence of sulfuric acid as a catalyst (2 wt % relative to GLY and MA) for 8 min at 120 °C under stirring. The resulting product was yellowish, homogeneous, and viscous. Although the reaction time was short, the desired product was successfully obtained. Longer reaction times led to solidification due to the formation of trisubstituted oligomers, which promote extensive cross-linking.

### Resin formulation

All formulations were composed of PPH and HEMA as a potential biobased reactive diluent, and 100 g of each resin were prepared. As the viscosity of PPH is high, this component was heated to 70 °C before HEMA addition, as already reported in the literature.^29^ The type 1 photoinitiator TPO was added to all formulations (3 wt % of monomeric mixture weight). Table 1 exhibits the composition of each resin, its name, and its biobased content (BC).

**Table 1:** Composition of the Photocurable Resins, Photoinitiator Amount, the Corresponding Biobased Content (BC), and Sustainable Formulation Score (SFS)

resin name	PPH (wt %)	HEMA (wt %)	TPO amount/wt %^a^	BC (%)	SFS
PPH30	30.0	70.0	3.0	97.0	45
PPH50	50.0	50.0	3.0	97.0	44
PPH70	70.0	30.0	3.0	97.0	43

a The percentage of photoinitiator used was calculated based on the total mass of monomers in the resin (PPH + HEMA).

### 3D Printing of PPH-Based Resins

The 3D printing was performed in an Elegoo Mars 2 Pro DLP 3D printer equipped with a 405 nm light source (3.8 mW cm^–2^), and all formulations were used in the printing process. The 3D-printed specimens were washed with isopropyl alcohol and postcured at room temperature with an Elegoo Wash & Cure Mercury Plus, for 10 min under UV irradiation (405 nm). The printing parameters, such as the exposure time and the layer thickness, were adjusted using Chitubox slicing software, as shown in Table 2. Furthermore, the PPH30 was used to produce a 3D-printed lattice structure with a height of 20 mm. The 3D-printed materials from PPH30, PPH50, and PPH70 were designated POL30, POL50, and POL70 respectively.

**Table 2:** Printing Parameters Used for all Resins

printing parameters	settings
layer height	0.05 mm
base layers amount	18
exposition time for base layers	50 s
exposition time for the rest of the layers	15 s
elevation after printing	5 mm

### Tuning Process for the 3D-Printed Materials

The materials were tuned by subjecting the 3D-printed specimens to a 120 min postcure process under UV light (365 nm, Kessil KSPR160, *E*_max_ = 399 mW cm–^2^) immediately after the first postcure (for 10 min at 405 nm). The distance between the light source and the specimens was maintained at 5 mm. The tuned materials were then characterized using thermal, mechanical, and spectroscopic techniques. The tuned materials were designated using the original name (e.g., POL30) appended with the second postcure time. For example, POL30 postcured for 120 min was designated POL30-120.

### Characterization

#### Biobased Content

The theoretical BC was calculated using eq 1,^30^ in which *m*_PPH_, *m*_HEMA_, and *m*_TPO_ are the masses in grams of each resin component. In this case, PPH was considered 100% biobased, as glycerol and maleic anhydride can potentially be produced from renewable resources.^31,32^ HEMA was also considered biobased,^33,34^ while TPO was considered a fossil-based compound.

1 $$BC\left(\%\right)=\left[\frac{\left(m_{PPH}+m_{HEMA}\right)}{\left(m_{PPH}+m_{HEMA}+m_{TPO}\right)}\right]\times 100$$

#### ^1^H NMR Analysis

The ^1^H NMR spectrum of PPH was acquired in an Agilent 400 MHz Premium Shield spectrometer. The sample was solubilized in deuterated water (D_2_O, 99.9% D, Sigma-Aldrich).

#### Mid-Infrared Spectroscopy Analysis (MIR) and Monomer Conversion

MIR spectra were obtained using a Bruker Vertex 70 FT-IR spectrometer with an attenuated total reflectance diamond crystal accessory. The measurements were performed in the range of 4000–400 cm^–1^ with 32 scans and a 4 cm^–1^ resolution.

Monomer conversion to polymer (MC %) was evaluated according to our previous work.^29^ The total area of the C=C bond bands (present in PPH and HEMA) at 1635 cm^–1^ (*A*_1635_) was measured before and after the printing process (and postcure processes for the tunable properties study). The spectra were normalized by the total area of the stretching band of methylene groups at 2956 cm^–1^ (*A*_2956_). eq 2 was used to determine the MC % values.

2 $$MC\%=\frac{{\left(\frac{A_{1635}}{A_{2956}}\right)}_{t=0}-{,\left(\frac{A_{1635}}{A_{2956}}\right)}_{t=x}}{{\left(\frac{A_{1635}}{A_{2956}}\right)}_{t=0}}\times 100$$

t = 0 refers to the resin before the printing, and *t* = *x* to the material obtained after the printing, or after a determined “*x*” postcure time.

#### Solid State Ultraviolet–visible (UV–Vis)

Solid UV–vis spectra were obtained in a PerkinElmer Lambda 1050 double-beam spectrophotometer. The 3D-printed square specimens (20 mm long, 20 mm wide, and 1 mm thick) used were printed from the PPH30 resin under the conditions described in Table 2.

#### Photoluminescence (PL)

Emission spectra were recorded with a Hitachi fluorescence spectrophotometer, model F-4500, with a 150 W xenon lamp as an excitation source. The same specimens utilized in the UV–vis analyses were excited at 365 nm with a slit of 2.5 nm during this analysis.

#### Simultaneous Thermogravimetry—Differential Thermal Analysis (TG–DTA)

Simultaneous TG–DTA analysis was performed in a STA 449 F3 equipment (Netzsch) using open α-alumina crucibles (200 μL), approximately 10 mg of sample, a temperature range of 30 to 800 °C, heating rate of 10 °C min^–1^, and dry air atmosphere (70 mL min^–1^).

#### Differential Scanning Calorimetry

DSC curves were acquired in a DSC1 Star^e^ (Mettler–Toledo). The following conditions were used: 5 mg of each sample, 40 μL closed aluminum crucible with a perforated lid, a flow rate of 50 mL min^–1^ of dry air, and a temperature range −10–100 °C with a heating rate of 10 °C min^–1^.

#### Dynamic Mechanical Analysis

Samples with dimensions of 20 mm length, 5 mm width, and 1 mm thickness were designed, 3D-printed, and analyzed in a Q800 apparatus (TA Instruments) after the tuning process. The temperature range used was −30 to 100 °C at a heating rate of 3 °C min^–1^ and a frequency of 1 Hz. The DMA curves provided the storage modulus (*E*′) and the tan-δ values. The glass transition temperature (*T*_g_) was determined from the temperature at the maximum loss factor (tan-δ).^35^ The tensile test (stress × strain curves) for each 3D-printed and tuned polymer was determined in the same DMA equipment with the following conditions: temperature of 25 °C, 3D-printed samples with dimensions of 20 mm length, 5 mm width, and 1 mm thickness, force ramp of 3 N min^–1^, and upper force limit of 18 N. It should be noted that the maximum elongation and maximum tensile strength values were determined at the point of maximum force, as the samples did not break under the experimental conditions performed.

The shape memory performance of the 3D-printed material after the tuning process (POL30-120) was evaluated by DMA analysis, as already reported in the literature.^36^ This analysis considers the respective shape fixity ratio (*R*_f_) and shape recovery ratio (*R*_r_). A sample with dimensions of 20 mm in length, 5 mm in width, and 1 mm in thickness was used for this. Four cycles were performed, and each cycle was composed of the following steps: (I) deformation: the specimen was equilibrated at *T*_g_ + 30 °C for 10 min, followed by a ramp force of 0.5 N min^–1^ up to 1.5 N. At the end of this ramp, the strain was recorded as ε_*m*_; (II) Cooling: in this step, a temperature ramp of 5 °C min^–1^ to 20 °C was performed, and the sample was equilibrated at this temperature for 30 min; (III) Fixing: the external force was removed according to a ramp force of 0.5 N min–1 to 0.001 N and an isothermal was kept for 30 min, and the strain was recorded as ε_*u*_ at the end of this stage; (IV) recovery: to access the recovery process, a temperature ramp of 5 °C min^–1^ to *T*_g_ + 30 °C was carried out and at the end of this process an isothermal was kept for 30 min, and the strain was recorded as ε_*f*_. Finally, the *R*_f_ and *R*_r_ values were obtained from eqs 3 and 4.

3 $$R_{f}\left(N\right)=\frac{\varepsilon_{u}\left(N\right)}{\varepsilon_{m}\left(N\right)}\times 100\%$$

4 $$R_{r}\left(N\right)=\frac{\varepsilon_{m}\left(N\right)-\varepsilon_{f}\left(N\right)}{\varepsilon_{m}\left(N\right)-\varepsilon_{f}\left(N-1\right)}\times 100\%$$

#### Hardness

The hardness was evaluated using an analogic Shore D durometer on 3D-printed specimens in the form of squares (60 mm × 60 mm × 30 mm) produced from each formulated resin.

## Results and Discussion

### Spectroscopy Properties and Sustainability Assessment

The occurrence of the reaction was verified by looking at the ^1^H NMR spectrum collected from the PPH (Figure S1). After reaction with MA, signals between 3.81 and 4.24 ppm are verified, and they can be associated with the hydrogens from the glycerol backbone in the polyester chain (Ha). Besides, the vinylic hydrogens (Hb) from the acrylic moiety are observed at 6.17 and 6.47 ppm. Lower-intensity signals observed between 4.25 and 4.42 ppm, as well as at 5.15 ppm, originate from a possible trisubstituted structure.^29^ These results confirm the production of α,β-unsaturated polyesters with potential for radical polymerization.

As glycerol is a renewable byproduct generated during biodiesel production, and considering that maleic anhydride and HEMA can potentially be produced from the oxidation of renewable furfural and from renewable methacrylic acid and ethylene glycol, respectively, a theoretical BC of 97% was obtained for all photocurable formulations.^31–34^ Considering that the same optimized conditions from our previous work were maintained for the synthesis of PPH,^29^ each repeating unit of this compound corresponds to the structure shown in Figure S2. Therefore, to provide a holistic assessment of the sustainability profile of the PPH-based formulations, the theoretical sustainable formulation score (SFS) for each resin was determined according to the procedure described by Maturi et al.^37^ As shown in Table 1, theoretical SFS values above 43 were obtained for the resins, indicating a moderate to good sustainability profile. This can be attributed to the potentially greener nature of the reagents used and the safer synthesis procedures employed in the production of each monomer. All values used to calculate the SFS values are presented in Table S1. These considerations in determining the theoretical BC and SFS highlight the importance of sustainable production processes in the polymer field.

The MIR spectra of the resins are presented in Figure 1a. The band at 1635 cm^–1^ is associated with C=C stretching, which is present in all formulations, varying only in intensity before and after polymerization. As polymerization occurs through chain growth, C=C bonds are consumed, and as expected, this is reflected in a decrease in the intensity of its respective stretching band in the MIR spectrum. According to our previous work, after the 3D printing and the first postcure process (for 10 min at 405 nm), it was observed that the polymers from all resins did not reach a complete monomer conversion (Table 3).^29^ As shown in Figure 1c, a complex-shaped lattice structure with a height of 20 mm was 3D-printed using PPH30, as this resin presented the best resolution results in a previous work.^29^ Therefore, although the MC % after the printing does not reach 100%, complex objects can be produced using this renewable resin.

**Figure 1:**
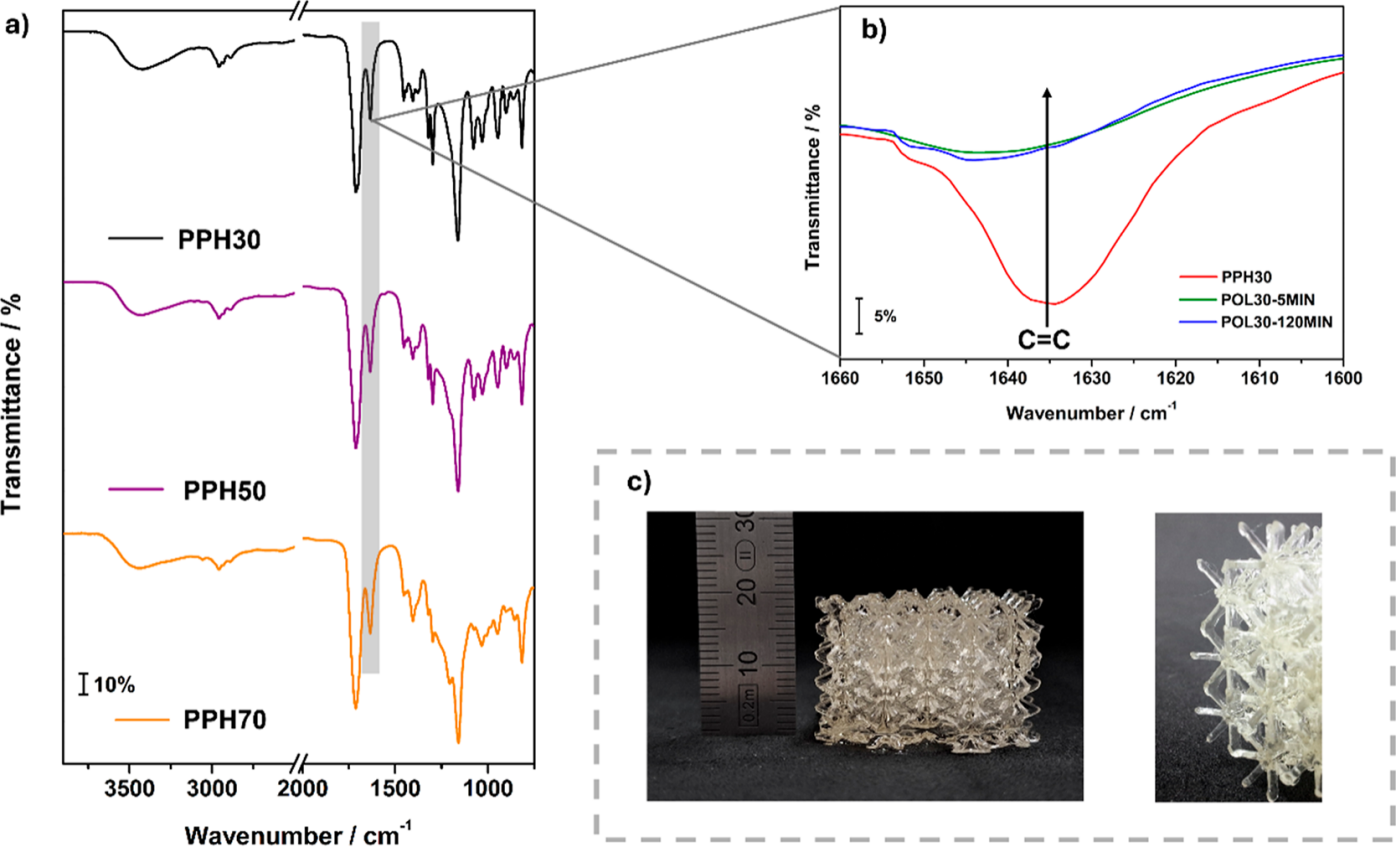
(a) MIR spectra for the resins and (b) magnification of MIR spectra for PPH30 and the 3D-printed polymer after 5 and 120 min of postcure under UV (365 nm). (c) 3D-printed lattice structure with 20 mm of height.

**Table 3:** Physical-Chemical Properties of all Tuned 3D-Printed Polymers Compared with Those Without the Second Post-cure

	after 120 min under UV			before the second post cure^29^		
	POL30-120	POL50-120	POL70-120	POL30	POL50	POL70
MC (%)	78.0	85.0	63.0	72	62	41
*T*_stability_ (°C)	120.7	134.7	127.5	98.4	97.6	95.7
*T*_g_ (°C)	62.6	---^b^	18.1	60.1	52.2	3.3
Tan-δ (°C)	56.5	54.1	16.0	61.0	50.2	9.7
maximum elongation (%)^a^	10.2	0.9	18.3	98.4	130.9	31.4
maximum tensile strength (MPa)^a^	2.7	2.8	1.2	0.4	0.3	0.3
hardness (shore D)	68	34	30	50	14	11

a The maximum elongation and maximum tensile strength values were determined at the point of maximum force, as the samples did not break under the experimental conditions performed.

b *T*_*g*_ was not detected by DSC.

Thus, the possibility of tuning the thermal and mechanical properties was explored by subjecting the 3D-printed materials from all resins to a second postcuring process under UV irradiation (365 nm) for 120 min after printing. In Figure 1b, a decrease in the band at 1635 cm^–1^ can be observed for the sample POL30-120 after 5 and 120 min of the second postcure. Table 3 displays the MC % values of the polymers after this postcure. This decrease was also observed in POL50-120 and POL70-120, depicted in Figures S3 and S4. Compared with the conversion after the printing (shown in Table 3), the conversion of POL30-120 increased by 6% after the second postcure, indicating residual polymerization. This value is lower than those presented by POL50-120 and POL70-120, which increased conversion by 23.0% and 22.0%, respectively, after the same process. The reduced chain mobility can explain the significantly lower conversion of POL30-120 compared to the other polymers. As monomer conversion increases, chain mobility decreases, leading to diffusion limitations in the propagation reaction and inhibiting further polymerization.^38^ Moreover, the higher MC % presented by POL50-120 may reflect a synergism between HEMA and PPH. In this case, HEMA contributes to reactivity due to the high content of active radicals. Simultaneously, PPH contributes to chain mobility, which allows for higher conversion. For POL70-120, the lower reactivity of PPH predominates, resulting in the lowest MC % after the second postcure.

### Thermal Properties

As polymers become more cross-linked, their thermal and mechanical properties tend to be affected.^38^ Thermogravimetric analysis (Figure 2a–c) showed that the increase in MC % after the postcure process affected the thermal stability of the polymers. Table 3 shows a significant rise in *T*_stability_ values for POL50-120 and POL70-120, with differences of 37.1 and 31.8 °C, respectively. For POL30, an increase of 14.4 °C was observed, related to the small change in MC % after the postcure of this material. Furthermore, there is no notable difference in the thermal profile, which exhibits three mass loss steps according to the TG and DTG curves. Exothermic events are verified in the DTA curve, and they mostly result from degradation processes.^29^ Complete TG-DTA data are available in Table S2.

**Figure 2:**
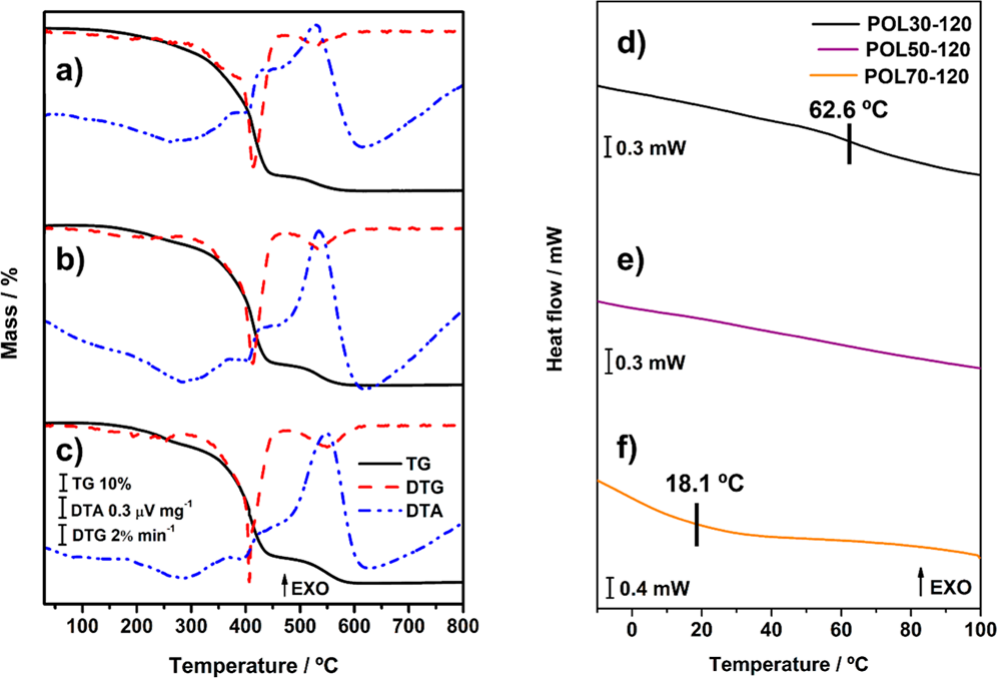
TG/DTG-DTA and DSC curves for (a,d) POL30-120, (b,e) POL50-120, and (c,f) POL70-120.

DSC curves of all tuned polymers are displayed in Figure 2d–f. Table 3 summarizes the observed *T*_g_ values. As expected, the *T*_g_ of POL30-120 remained relatively unchanged after the tuning process, likely due to the minimal variation in its monomer conversion (MC %). While the DSC curve for POL50-120 did not exhibit a discernible glass transition event, DMA analysis confirmed its presence. Finally, POL70-120 presented a *T*_g_ increase of 8.4 °C, which can be attributed to the increase in MC % after the tuning process.

### Mechanical Properties

Relaxation curves (Figure 3a) and the data in Table 3 show that the tan-δ temperature did not change significantly for POL30-120 and POL50-120 after the postcure process. At the same time, POL70-120 exhibited the largest variation in tan-δ temperature, as already observed by DSC. Although no significant changes are observed in the α-transitions, the secondary transitions (or β-transitions) were affected after the postcure process. For POL30-120 and POL50-120, the maximum tan-δ peak intensity from the β-transitions was significantly reduced compared with that observed previously^29^ (before the tuning process). It is suggested that this reduction in intensity is attributed to the increased conversion, which leads to a decrease in the concentration of free linear PPH segments. Consequently, the free volume decreases, resulting in the observed reduction in tan-δ peak intensity.^39^ Moreover, the shift in *T*_g_ for POL70-120 indicates that this material also has secondary transitions at −14.4 °C, as expected, since these materials are semi-interpenetrating polymer networks.

**Figure 3:**
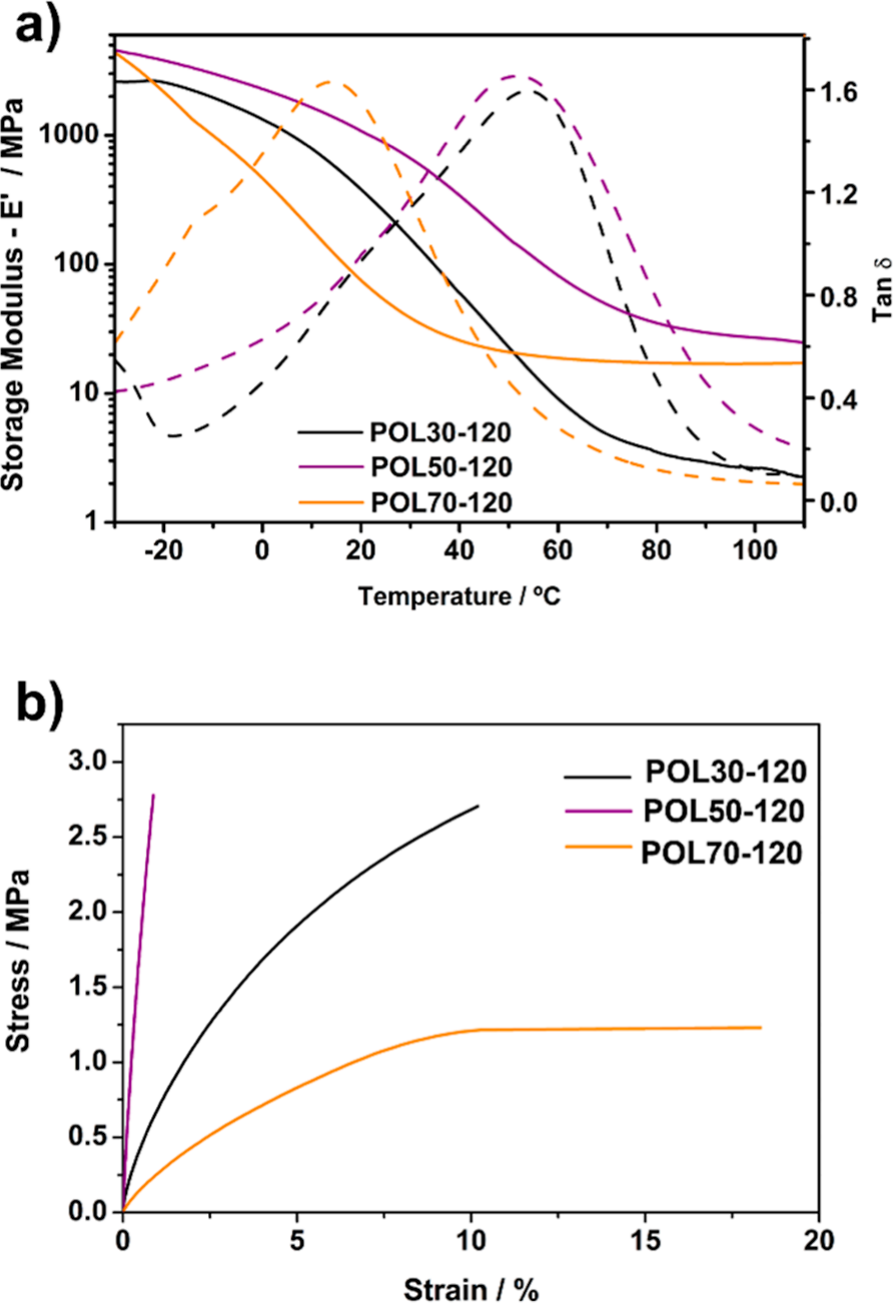
(a) Relaxation and (b) stress–strain curves for the tuned 3D-printed polymers.

Increased monomer conversion resulted in more cross-linked polymers, leading to a significant decrease in maximum elongation and an increase in maximum tensile strength for all polymers, as shown in Figure 3b and Table 3. POL50-120 exhibited the highest maximum tensile strength and the lowest strain, likely due to the substantial increase in MC %. POL70-120 now exhibits the highest strain, which may be attributed to the high concentration of PPH in the polymer network. Thus, after the postcure process, all polymers became more rigid.

After the tuning process, all polymers’ hardness (displayed in Table 3) increased. While the increase in MC % appears to be a primary factor, this property is not exclusively dependent on them. The components of the formulation directly influence hardness; for example, although POL50-120 had a higher MC % than POL30-120, it exhibited lower hardness. This suggests that increasing the PPH content in the formulation leads to softer materials, even when high MC % values are achieved. Thus, HEMA appears to be a hardening agent, while PPH is a softening counterpart.

### Luminescent Properties

During the postcure process (for tuning the material) under UV irradiation, all polymers were observed to become luminescent. This behavior was not observed in the polymers before the tuning process, as shown in Figures 4a and S5. Thus, it was suggested that, besides UV exposure interfering with MC % values, other chemical characteristics of the material may be altered. Then, to investigate and explain this behavior, solid-state UV–vis, photoluminescence, and MIR analyses were performed using the 3D-printed polymer from PPH30 (called POL30) as the subject of study. Solid UV–vis spectra were collected for this material before (after printing and the first postcure) and during the second postcure process at various times (1, 5, 15, 30, 60, and 120 min), as shown in Figure 4b.

**Figure 4:**
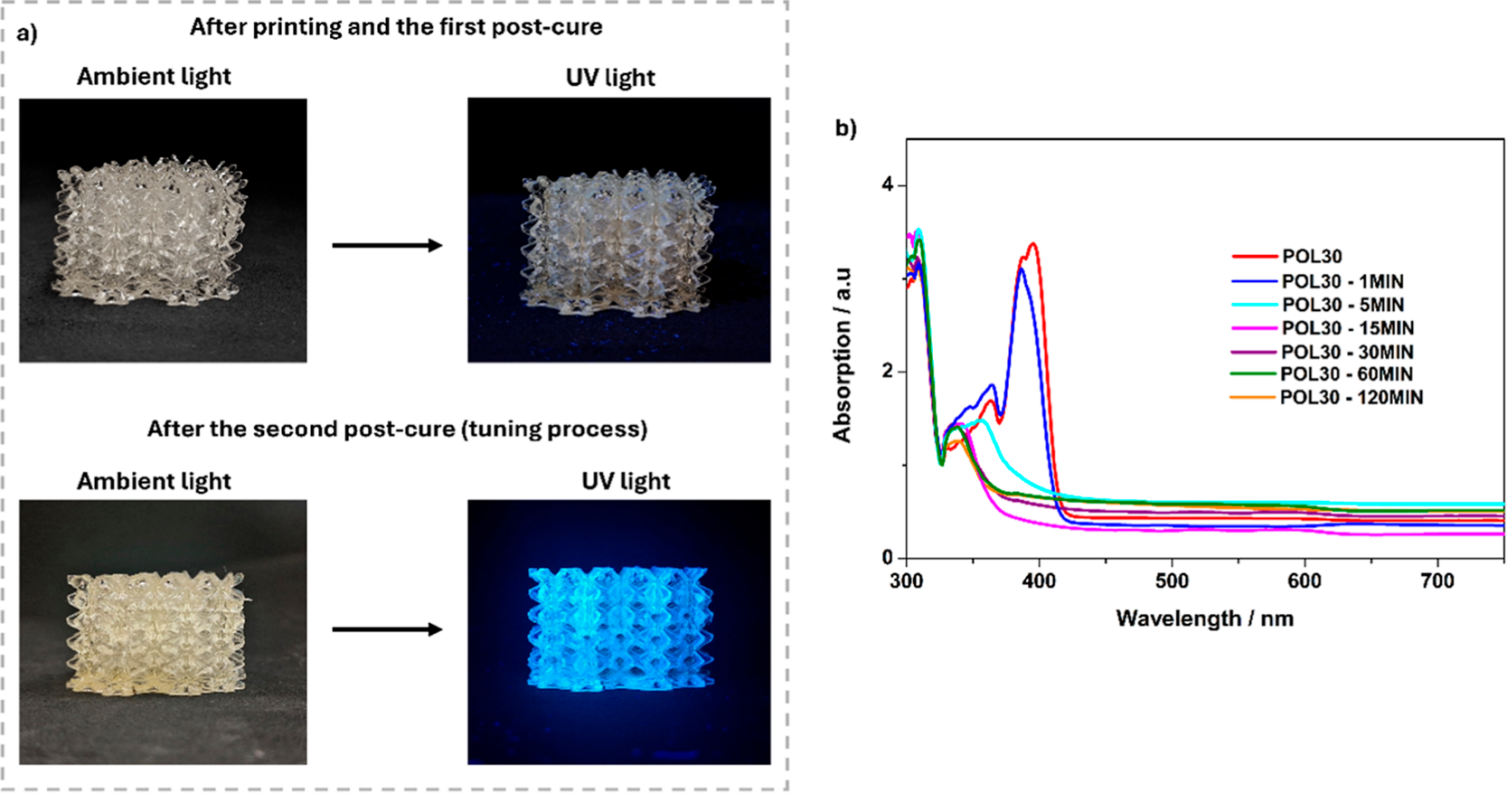
(a) 3D-printed lattice structure from PPH30 under ambient and UV light before and after the second postcure process. (b) Solid UV–vis spectra for POL30, collected before (after printing and the first postcure) and during the second postcure process at different times (1, 5, 15, 30, 60, and 120 min).

The UV–vis absorption spectrum shows an intense absorption band with a maximum absorption peak (λ_max_) at 394 nm, a shoulder at 388 nm, and a less intense band at 364 nm for the polymer before the tuning process. After 5 min of postcure, the highest intensity band (at 394 nm) was no longer present, and the band at 364 nm shifted to λ_max_ = 354 nm, disappearing after 15 min. Additionally, after 5 min, an increase in the intensity of the band at 338 nm was observed, persisting until the end of the postcure (120 min). Based on these results and qualitative observations (Figure 5a–c), it was concluded that the luminescence process begins after 5 min and is still visible after 120 min. Therefore, samples of POL30 and the polymers postcured for 5 and 120 min (POL30-5 and POL30-120, respectively) were used for the subsequent analyses.

**Figure 5:**
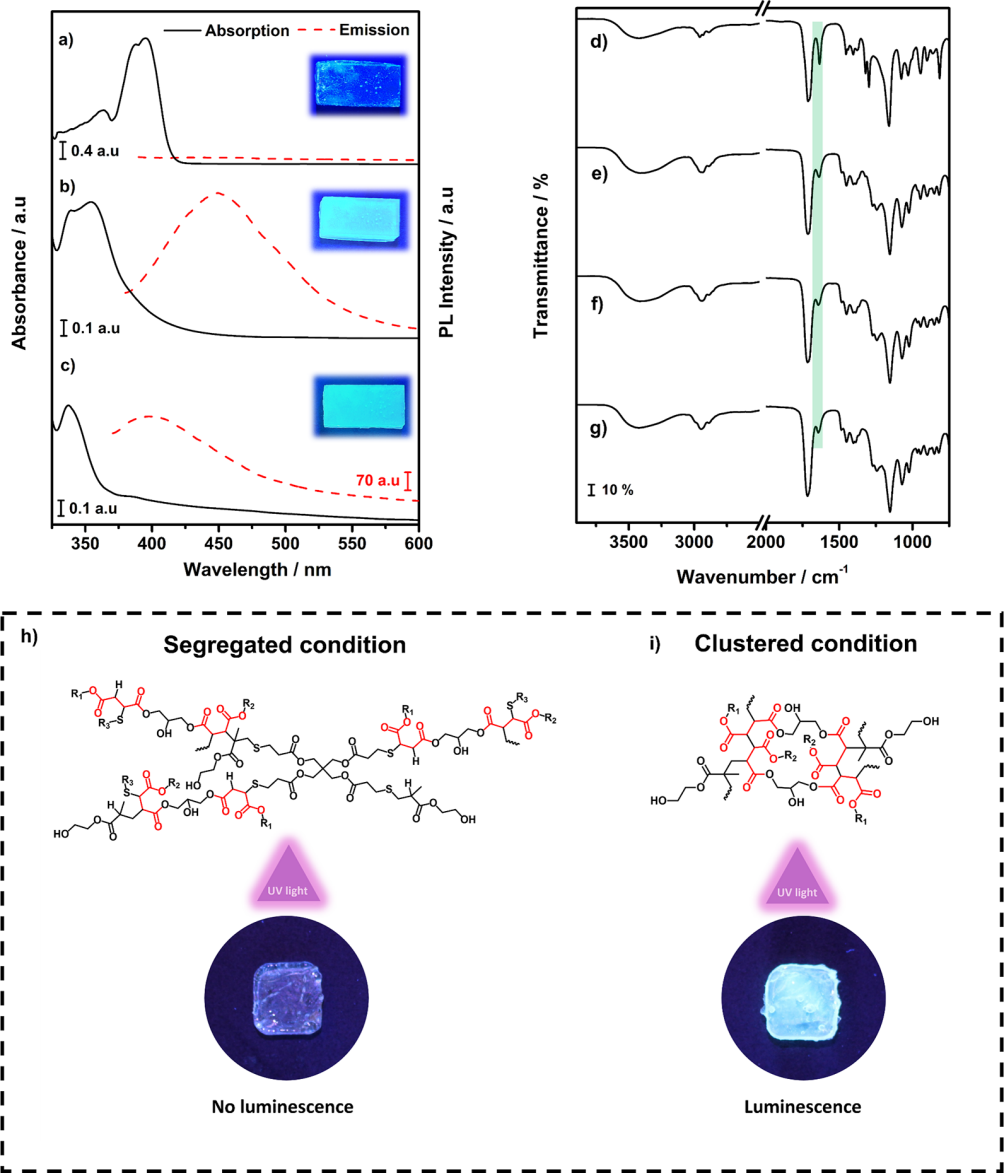
Relationship between UV–vis, fluorescence spectra, and corresponding images of (a) POL30, (b) POL30-5, and (c) POL30-120 under UV light (365 nm). MIR spectra for (d) PPH30, (e) POL30, (f) POL30-5, and (g) POL30-120, highlighting double bond consumption over time. Polymers obtained under UV light (365 nm) and structural proposal for (h) the segregated form of POL30-PT3M and (i) the clustered form of POL30-C.

Figure 5 shows a comparison of solid UV–vis absorption and photoluminescence spectra for POL30, POL30-5, and POL30-120. In the emission spectra, no signal is observed for POL30, while for POL30-5, a band with λ_max_ = 448 nm is observed, resulting in a Stokes shift of 94 nm (considering the band at 354 nm in the solid absorption spectrum), confirming fluorescence. After 120 min, emissions are still identifiable; however, the intensity of the emission band has decreased, and a shift to λ_max_ = 398 nm is observed. This shift in the emission profile of POL30-120 to lower wavelengths may be associated with the band at 338 nm in the absorption spectrum, resulting in a Stokes shift of 60 nm.

These results indicate that UV exposure decreased the conjugation of chromophore groups, such as the double bonds, leading to absorptions at lower wavelengths (as observed in the UV–vis spectra) due to an increase in the band gap. Furthermore, this reduction in conjugation correlates with the observed fluorescence property, its intensity, and emission, which are reduced and shifted to lower wavelengths by prolonged exposure. The consumption of chromophore groups was confirmed by MIR analyses in Figure 5d,g, which show an MC % = 77% after 5 min of postcure (indicating that 5% of double bonds were consumed), increasing to 78% after 120 min (Table 3). Although the MC % value did not change significantly after 120 min, the material’s color changed from transparent to yellow.

Based on these findings, it is suggested that after 5 min of postcure, fluorescence is achieved through polymerization-induced emission (PIE). PIE is a process in which luminescence is observed in nonconjugated structures after a certain degree of polymerization. Polymers that fit this description are called nonconjugated luminescent polymers (NCLPs). In this case, as polymerization progresses, clusters are formed, and due to the aggregation-induced emission (AIE) effect, the material becomes an NCLP. AIE-based fluorescence in maleic anhydride-based polymers can occur due to the clustering of locked carbonyl groups when these groups are nearby, causing orbital overlapping, resulting in a low-lying LUMO state. Thus, when polymerization advances, it shortens the distances between carbonyl groups, producing clusters with delocalized nonbonding electrons for easier intra- and inter- *n* → π* interactions. In such interactions, the lone pair (*n*) of one carbonyl group overlaps with the empty π* orbital of another carbonyl, leading to orbital mixing, which creates low-lying LUMO states. The resulting reduction in HOMO–LUMO gap allows radiative decay in the near-UV or visible region. At the same time, the polymerization-caused clustering restricts intramolecular motions and vibrational degrees of freedom, which suppresses nonradiative relaxation pathways. Consequently, the excited electron trapped in this stabilized, delocalized π* state can relax radiatively back to the ground state, producing luminescence.^40–45^ In our case, it is suggested that, as PPH has numerous carbonyl groups along its chain, and after reaching a certain degree of polymerization, it becomes luminescent due to the PIE/AIE effect. This hypothesis is supported by similar results described in the literature for carbonyl-rich polymers.^41–45^

In addition, any change was observed in the carbonyl band (intensity or displacement) in the MIR spectrum of POL30-120 (Figure 5g), nor in the temperature of thermal stability (TG/DTG-DTA—Figure 2a). Therefore, the color change observed under visible light was attributed to the PIE effect.^41^

**Figure 6:**
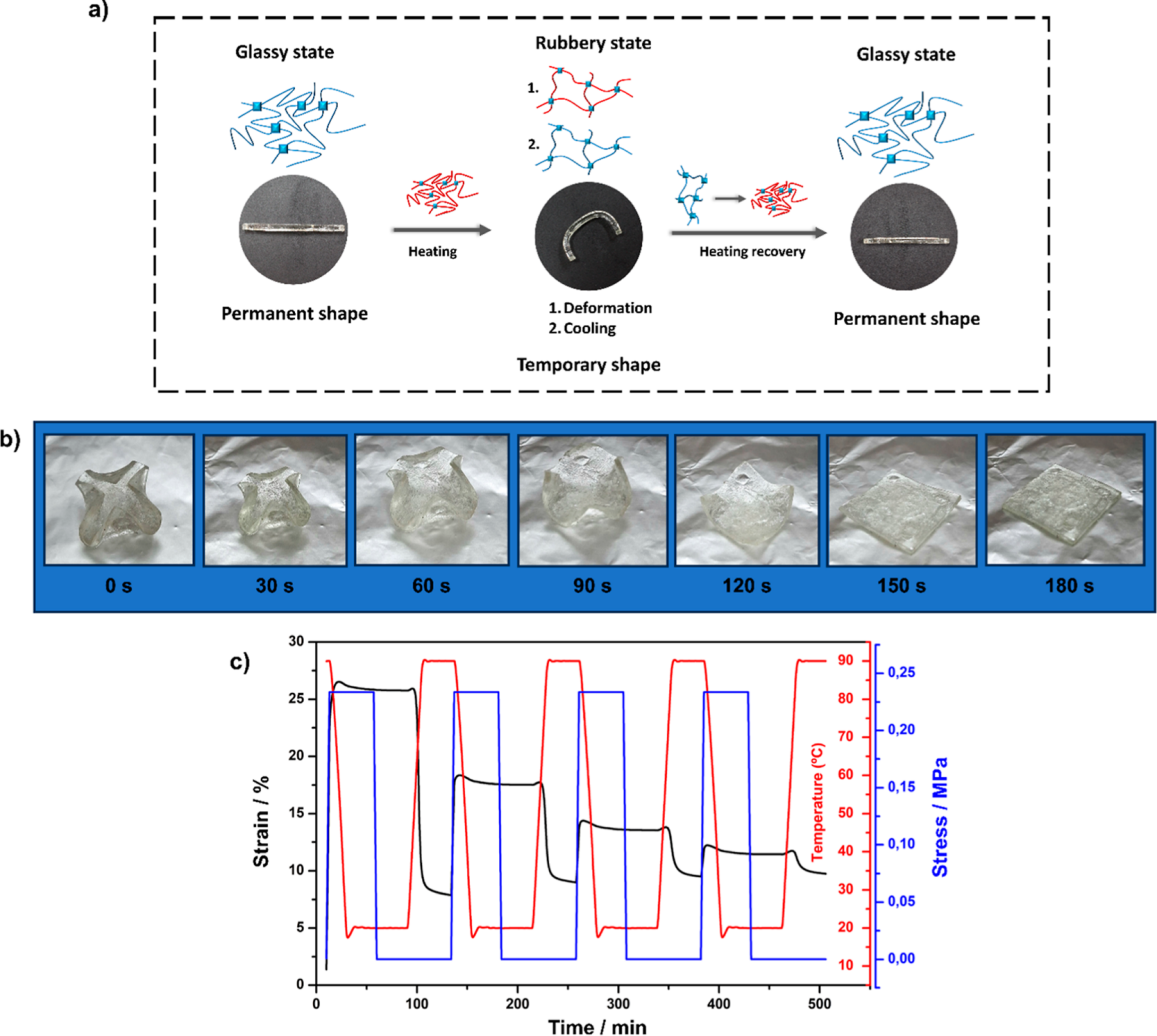
(a) Cycles of stress, strain, and temperature with time recorded by DMA during shape memory testing of POL30-120. (b) Qualitatively observed shape memory behavior for POL30-120 after heating at 84 °C. (c) Scheme of molecular changes during the shape memory process and qualitatively SMP behavior of POL30-120 after heating, deformation, cooling, and heating recovery.

As described by Zhou and others,^41^ the AIE effect can be confirmed by segregating the carbonyl groups using a long-chain comonomer as a segregating agent, which results in the absence (or reduction) of luminescence. Therefore, PPH30 and PPH30 containing 40 mol % (relative to the C=C amount in PPH30) of the polythiol pentaerythritol tetrakis­(3-mercaptopropionate) (PT3M) as the segregating agent were photopolymerized under UV light (365 nm) for 5 min. With the addition of PT3M in the formulation, polymerization occurs via a thiol–ene (or thiol-Michael) mechanism.^46^ This is confirmed by the double bond consumption observed in the MIR spectra collected before and after polymerization (Figure S6), resulting in an MC % value of 83%. Furthermore, as expected, the polymer obtained from the mixture of PPH30 and PT3M (designated POL30-PT3M and shown in Figure 5h) exhibited no qualitatively observable fluorescence due to the segregation of carbonyl groups. In contrast, the clustered polymer from only PPH30 (called POL30-C) is luminescent, confirming the AIE and, consequently, the PIE effect. Besides, a complex structure was 3D-printed from PPH30, and the PIE effect was recorded during the second postcuring process, as shown in Video S1. The video was recorded for 120 min and accelerated 270 times, clearly showing the evolution of luminescence.

### Shape Memory Properties

Within the field of smart materials, shape memory polymers (SMPs) are of particular interest due to their ability to return to their initial (or permanent) shape from a temporary shape after an external stimulus, such as light, magnetism, electricity, or heat. To induce this behavior, the permanent shape must first be stimulated (e.g., by heating) and then deformed above the transition temperature (*T*_transition_), such as the *T*_g_, resulting in a temporary shape that can be fixed upon cooling. Subsequently, the material can return to its original shape by reheating above the *T*_transition_. Figure 6a shows a schematic of SMP behavior based on our findings and phase transition theory,^47^ which explains that an SMP is composed of two structural components: a frozen phase and an active phase. When the enthalpy energy of the material changes, the frozen phase is stretched or rotated to prevent changes in the internal structure, thus becoming responsible for shape recovery (the material’s memory).^47^

Conversely, when the polymer changes from a glassy to a rubbery state, some segments of the frozen phase transform into the active phase, allowing conformational movements such as molecular rotation, elongation, and compression.^47^ In the glassy state, the frozen phase is dominant. However, when the temperature rises above *T*_g_ (or another *T*_transition_) through heating, the material enters a rubbery state where the active phase becomes dominant.^47^ In our case, the frozen phase consists of the cross-linked points (blue box in the scheme), primarily from cross-linked HEMA/PPH or just PPH, and the active phase comprises the other segments of the polymer structure, such as the glycerol backbone chain of PPH or segments of HEMA that, while attached, are not cross-linked. After heating and deformation, the active phase changes from a twisted form to a more ordered form (represented by the long red lines in the scheme), with the conformational changes being stored in the active phase as internal stress.^46^ During the cooling (or fixing) process, the active phase becomes frozen again, and the internal stress is insufficient to provide the enthalpy required for shape recovery, thus maintaining the temporary shape. However, when the temperature rises above *T*_g_, the stored energy is released due to the action of internal stress and molecular motion, allowing the active phase to return to its initial state and recover the permanent shape.

POL30-120 was selected for shape memory polymer (SMP) testing due to its high room-temperature hardness and rigidity after the second postcure. The SMP behavior of this 3D-printed material was qualitatively assessed and quantitatively measured via dynamic mechanical analysis (DMA). Figure 6a,b show that SMP behavior is observed for 3D-printed POL30-120 objects with different shapes, and that approximately 180 s is necessary for recovery above the *T*_g_ temperature. Meanwhile, Figure 6c shows the results of four sequential shape memory cycles performed on POL30-120, with data summarized in Table 4. The shape fixity ratio (*R*_f_), calculated using eq 3, was 97% in the first cycle and 94% in the final two cycles, indicating high shape fixity. The shape recovery ratio (*R*_r_), calculated using eq 4, progressively increased with each cycle, reaching 91% by the fourth cycle.

**Table 4:** Shape Fixation and Recovery Ratios (*R*_f_ and *R*_r_) in Four Continuous Shape Memory Cycles for POL30-120

cycle	*R*_f_/ %	*R*_r_/ %
cycle 1	97	70
cycle 2	96	89
cycle 3	94	90
cycle 4	94	91

During the SMP test, with a constant applied force of 1.5 N in each cycle, a decrease in strain was observed with each successive cycle. This can be attributed to stiffening. MIR spectra obtained after the test (Figure S7) indicated an MC % of 99.6%, corresponding to a 21.6% increase relative to the polymer before testing. Thus, it can be concluded that heating promotes residual polymerization, increasing the cross-linking of PPH segments. This reduces the active phase, leading to a decrease in material strain (under constant force) during cycling. However, according to eqs 3 and 4, the material retained its SMP behavior. While its shape-fixing capacity was not significantly affected, the formation of additional cross-linked points (frozen phases) increased its capacity for permanent shape recovery, as expected for thermosets.^46^ As shown qualitatively in Video S2, the recovery capacity of POL30-120 is kept within four shape memory cycles, confirming the 4D printing of a renewable material.

## Conclusion

In this work, renewable thermosets were 3D-printed from potentially biobased resins obtained from glycerol and maleic anhydride, which presented a theoretical biobased content of 97% and theoretical sustainable formulation scores above 43. The materials were then submitted to a second postcure process by employing UV (365 nm) irradiation, and several properties were improved or even developed. After this tuning process, the materials exhibited enhanced thermal and mechanical performance. In addition, a shape-memory behavior was observed in POL30-120, representing an advance toward greener 4D printing of materials with tunable properties. Furthermore, luminescent behavior was achieved in all materials after the second postcure, making them suitable for on-demand applications.

A substantial increase in the thermal stability of the thermosets was also observed, along with an increase in the rigidity of the materials, with a displacement of the glass transition temperature, the maximum tensile strength, and hardness to high values after the tuning process.

The fluorescence observed in these materials after 5 min of postcure was investigated, and PIE as well as AIE effects were identified as the underlying mechanisms for this behavior. The shape memory performance of POL30-120 was evaluated, demonstrating high shape fixing and recovery properties after four cycles, likely due to the presence of both cross-linked and non-cross-linked segments in the material.

## Supplementary Material







## Data Availability

The authors confirm that all relevant data are included in the article.

## Notes

^1^H NMR spectra; MIR spectra; information about the TG-DTA analyses; equations for Sustainable Formulation Score calculation; pictures of all polymers before and after the tuning process; qualitative test for shape-memory behavior (PDF)
Video S1 (MP4)
Video S2 (MP4)

## References

[1] Alarcon R. T., dos Santos G. I., Gaglieri C., de Moura A., Cavalheiro E. ´. T. G., Bannach G. (2024). Lipidic Biomass as a Renewable Chemical
Building Block for Polymeric Materials. Chem.
Comm.

[2] Bagheri A., Jin J. (2019). Photopolymerization
in 3D Printing. ACS Appl.
Polym. Mater..

[3] Chin K. C. H., Cui J., O’Dea R. M., Epps T. H., Boydston A. J. (2023). Vat 3D
Printing of Bioderivable Photoresins – Toward Sustainable and
Robust Thermoplastic Parts. ACS Sustain. Chem.
Eng..

[4] Kutz, M. Smart Materials. In Mechanical Engineers’ Handbook: Materials and Mechanical Design, 3rd ed.; Kutz, M. , Ed.; John Wiley and Sons, 2006.

[5] Karunakar K. K., Cheriyan B. V., Anandakumar R., Murugathirumal A., Senthilkumar A., Nandhini J., Kataria K., Yabase L. (2025). Stimuli-Responsive
Smart Materials: Bridging the Gap between Biotechnology and Regenerative
Medicine. Bioprinting.

[6] Bahl S., Nagar H., Singh I., Sehgal S. (2020). Smart Materials
Types,
Properties and Applications: A Review. Mater.
Today: Proc..

[7] Chatterjee S., Chi-leung Hui P. (2019). Review of
Stimuli-Responsive Polymers in Drug Delivery
and Textile Application. Molecules.

[8] Xu M. M., Liu R. J., Yan Q. (2018). Biological Stimuli-Responsive
Polymer
Systems: Design, Construction and Controlled Self-Assembly. Chin. J. Polym. Sci..

[9] Wells C. M., Harris M., Choi L., Murali V. P., Guerra F. D., Jennings J. A. (2019). Stimuli-Responsive Drug Release from
Smart Polymers. J. Funct. Biomater..

[10] Rao N. V., Ko H., Lee J., Park J. H. (2018). Recent Progress and Advances in Stimuli-Responsive
Polymers for Cancer Therapy. Front. Bioeng.
Biotechnol..

[11] Wei H., Cui J., Lin K., Xie J., Wang X. (2022). Recent Advances in
Smart Stimuli-Responsive Biomaterials for Bone Therapeutics and Regeneration. Bone Res..

[12] Hoque J., Sangaj N., Varghese S. (2019). Stimuli-Responsive Supramolecular
Hydrogels and Their Applications in Regenerative Medicine. Macromol. Biosci..

[13] Zhou Q., Zhang L., Yang T. H., Wu H. (2018). Stimuli-Responsive
Polymeric Micelles for Drug Delivery and Cancer Therapy. Int. J. Nanomedicine.

[14] Shen Z., Chen F., Zhu X., Yong K.-T., Gu G. (2020). Stimuli-Responsive
Functional Materials for Soft Robotics. J. Mater.
Chem. B.

[15] Panda S., Hajra S., Rajaitha P. M., Kim H. J. (2023). Stimuli-Responsive
Polymer-Based Bioinspired Soft Robots. Micro
nano syst. lett..

[16] Kim S., Lee S. N., Melvin A. A., Choi J. W. (2024). Stimuli-Responsive
Polymer Actuator for Soft Robotics. Polymers.

[17] Chen J., Virrueta C., Zhang S., Mao C., Wang J. (2024). 4D Printing:
The Spotlight for 3D Printed Smart Materials. Mater. Today.

[18] Tariq A., Arif Z. U., Khalid M. Y., Hossain M., Rasool P. I., Umer R., Ramakrishna S. (2023). Recent Advances
in the Additive Manufacturing
of Stimuli-Responsive Soft Polymers. Adv. Eng.
Mater..

[19] Warner J. J., Wang P., Mellor W. M., Hwang H. H., Park J. H., Pyo S.-H., Chen S. C. (2019). 3D Printable
Non-Isocyanate Polyurethanes
with Tunable Material Properties. Polym. Chem..

[20] Lantean S., Barrera G., Pirri C. F., Tiberto P., Sangermano M., Roppolo I., Rizza G. (2019). 3D Printing
of Magnetoresponsive
Polymeric Materials with Tunable Mechanical and Magnetic Properties
by Digital Light Processing. Adv. Mater. Technol..

[21] Sachyani
Keneth E., Lieberman R., Rednor M., Scalet G., Auricchio F., Magdassi S. (2020). Multi-Material 3D Printed Shape Memory
Polymer with Tunable Melting and Glass Transition Temperature Activated
by Heat or Light. Polymers.

[22] Ma Q., Ren G., Xu K., Ou J. Z. (2021). Tunable Optical Properties of 2D
Materials and Their Applications. Adv. Opt.
Mater..

[23] Tian W., Zhang J., Yu J., Wu J., Zhang J., He J., Wang F. (2018). Phototunable Full-Color
Emission of Cellulose-Based
Dynamic Fluorescent Materials. Adv. Funct. Mater..

[24] Gao S., Tang G., Hua D., Xiong R., Han J., Jiang S., Zhang Q., Huang C. (2019). Stimuli-Responsive
Bio-Based Polymeric Systems and Their Applications. J. Mater. Chem. B.

[25] Constant E., King O., Weems A. C. (2022). Bioderived 4D Printable Terpene Photopolymers
from Limonene and β-Myrcene. Biomacromolecules.

[26] Brooks S., Merckle D., Weems A. C. (2023). 4D Photopolymers
Derived From Ring-Opening
Copolymerization of Cyclic Anhydrides and Limonene Oxide. ACS Sustain. Chem. Eng..

[27] Li B., Bartolini Torres G., Martin B., Taylor N., Barbu E., Christie A., Heise A. (2025). Polycaprolactone–Itaconic
Acid Resins for Additive Manufacturing of Environmentally Degradable
3D and 4D Materials by Thiol-Ene Photopolymerization. Macromolecules.

[28] THE 17 GOALS Sustainable Development. https://sdgs.un.org/goals (accessed 10 15, 2025).

[29] dos
Santos G. I., Gaglieri C., Alarcon R. T., de Moura A., dos Santos F. B., Bannach G. (2025). Glycerol and Maleic Anhydride-Based
Acrylic Polyester: A Solution for Greener Photocurable Resins for
3D Printing of Renewable Materials. ACS Sustain.
Chem. Eng..

[30] Alarcon R. T., Gaglieri C., Bannach G., Cavalheiro E. ´. T. G. (2024). Greener Preparation of a Flexible Material Based on Macaw Palm Oil
Derivatives and CO2. Green Chem..

[31] Kholif A. E. (2019). Glycerol
Use in Dairy Diets: A Systemic Review. Anim.
Nutr..

[32] Lan J., Chen Z., Lin J., Yin G. (2014). Catalytic Aerobic Oxidation
of Renewable Furfural to Maleic Anhydride and Furanone Derivatives
with Their Mechanistic Studies. Green Chem..

[33] Le
Nôtre J., Witte-van Dijk S. C.
M., van Haveren J., Scott E. L., Sanders J. P. M. (2014). Synthesis of Bio-based Methacrylic
Acid by Decarboxylation of Itaconic Acid and Citric Acid Catalyzed
by Solid Transition-metal Catalysts. ChemSusChem.

[34] Wang Y., Xian M., Feng X., Liu M., Zhao G. (2018). Biosynthesis
of Ethylene Glycol from D-Xylose in Recombinant Escherichia Coli. Bioengineered.

[35] Dong J., Liu B., Ding H., Shi J., Liu N., Dai B., Kim I. (2020). Bio-Based Healable Non-Isocyanate
Polyurethanes Driven by the Cooperation
of Disulfide and Hydrogen Bonds. Polym. Chem..

[36] Zhao L., Yu R., He Y., Zhang M., Tian F., Wang L., Zhao Y., Huang W. (2024). 3D Printed Epoxy/Acrylate Hybrid
Polymers with Excellent Mechanical and Shape Memory Properties via
UV and Thermal Cationic Dual-Curing Mechanism. Addit. Manuf..

[37] Maturi M., Locatelli E., Sanz de Leon A., Comes Franchini M., Molina S. I. (2025). Sustainable Approaches
in Vat Photopolymerization:
Advancements, Limitations, and Future Opportunities. Green Chem..

[38] Young J. S., Kannurpatti A. R., Bowman C. N. (1998). Effect of Comonomer Concentration
and Functionality on Photopolymerization Rates, Mechanical Properties
and Heterogeneity of the Polymer. Macromol.
Chem. Phys..

[39] Alyes N. M., Gómez Ribelles J. L., Gómez Tejedor J. A., Mano J. F. (2004). Viscoelastic Behavior
of Poly (Methyl Methacrylate)
Networks with Different Cross-Linking Degrees. Macromolecules.

[40] Liu B., Zhang H. K., Liu S. J., Sun J. Z., Zhang X. H., Tang B. Z. (2020). Polymerization-Induced Emission. Mater. Horiz..

[41] Zhou X., Luo W., Nie H., Xu L., Hu R., Zhao Z., Qin A., Tang B. Z. (2017). Oligo (Maleic
Anhydride) s: A Platform for Unveiling
the Mechanism of Clusteroluminescence of Non-Aromatic Polymers. J. Mater. Chem. C.

[42] Chen X., Liu X., Lei J., Xu L., Zhao Z., Kausar F., Xie X., Zhu X., Zhang Y., Yuan W. Z. (2018). Synthesis, Clustering-Triggered
Emission, Explosive Detection and Cell Imaging of Nonaromatic Polyurethanes. Mol. Syst. Des. Eng..

[43] Newberry R. W., Raines R. T. (2014). A Key N→π* Interaction in N-Acyl Homoserine
Lactones. ACS Chem. Biol..

[44] Zhao Z., Zhang H., Lam J. W. Y., Tang B. Z. (2020). Aggregation-Induced
Emission: New Vistas at the Aggregate Level. Angew. Chem., Int. Ed..

[45] Chen Y. J., Pu M. Q., Wu L. T., Sun X. L., Wan W. M. (2023). Nucleophilic
Substitution Polymerization-Induced Emission of 1,3-Dicarbonyl Compounds
as a Versatile Approach for Aggregation-Induced Emission Type Non-Traditional
Intrinsic Luminescence. Chin. J. Chem..

[46] Yan S., Zhang F., Luo L., Wang L., Liu Y., Leng J. (2023). Shape Memory Polymer
Composites: 4D Printing, Smart Structures, and
Applications. Research.

[47] Xia Y., He Y., Zhang F., Liu Y., Leng J., Xia Y. (2021). A Review of
Shape Memory Polymers and Composites: Mechanisms, Materials, and Applications. Adv. Mater..

