# Supplementary material for: 4D Printing of Renewable Materials Derived from Glycerol and Maleic Anhydride with Tunable Thermal, Mechanical, and Fluorescent Properties
Source: ACS Omega. 2026 Apr 8;11(15):23490–500. doi: 10.1021/acsomega.6c01077 (PMC13103836; doi:10.1021/acsomega.6c01077)
Supplement: Supplementary file 1 [file ao6c01077_si_001.pdf]

# 4D Printing of Renewable Materials Derived from Glycerol and Maleic Anhydride with Tunable Thermal, Mechanical, and Fluorescent Properties

## Supporting information

Gabriel I. dos Santos [a], Caroline Gaglieri [a], Rafael T. Alarcon [b], Aniele de Moura [c], Fernanda B. dos Santos [a], Gilbert Bannach [a*]

[a] Universidade Estadual Paulista (UNESP), Faculdade de Ciências, Bauru, 17033-260, Brazil

[b] Universidade de São Paulo (USP), Instituto de Química de São Carlos, São Carlos, 13566-590, Brazil

[c] Universidade Federal de São Carlos (UFSCar), Centro de Ciências Exatas e de Tecnologia, São Carlos, 13565-905, Brazil

* E-mail: gilbert.bannach@unesp.br

Number of Pages: 8

Number of Figures: 7

Number of Tables: 2

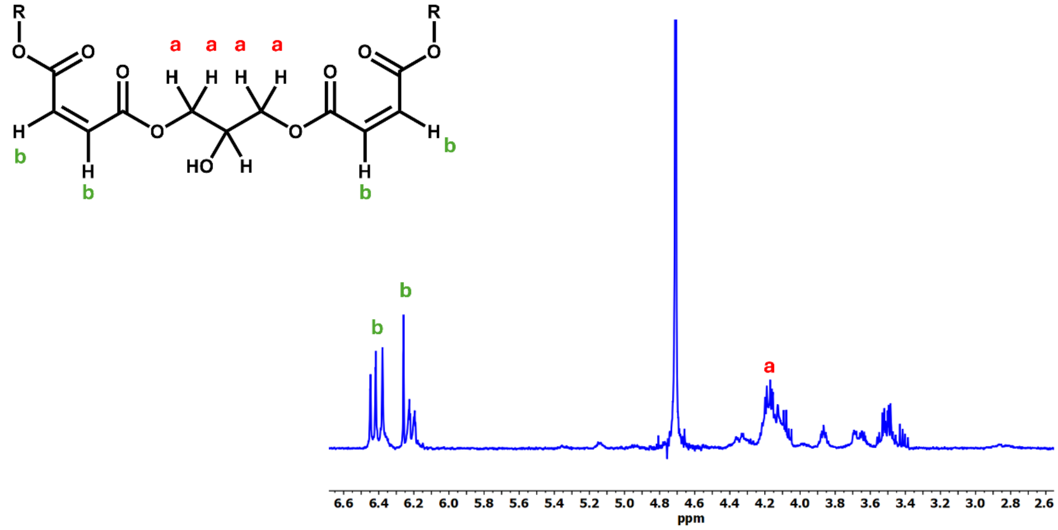


**Figure S1.** $^{1}$H-NMR spectrum of PPH.

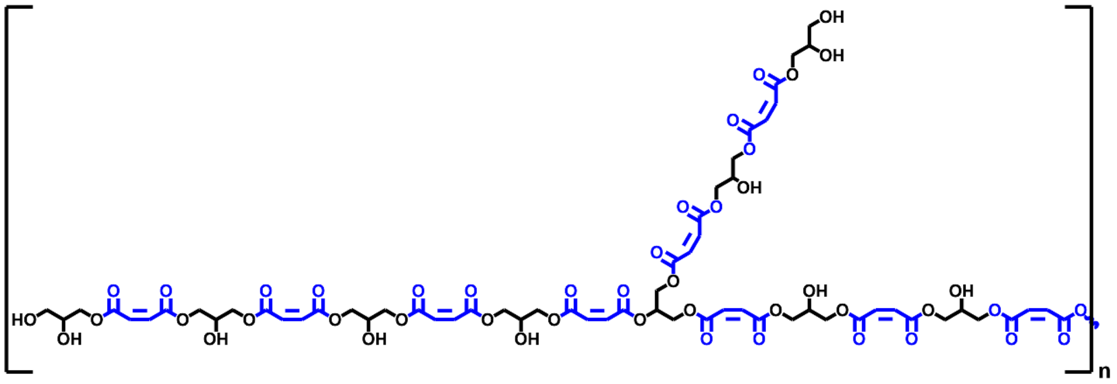


**Figure S2.** Structural unit of PPH.

**Sustainable formulation score calculation.** The sustainable formulation score (SFS) was calculated according to Eq. S1(see ref. 37).

$$\boldsymbol{SFS} = \mathbf{100} \times \boldsymbol{F_{EoL}} \times \sum_{i=1}^{n}(\boldsymbol{w_i} \times \boldsymbol{BCC_i} \times \boldsymbol{F_{syn,i}}) \tag{S1}$$

$\boldsymbol{F_{EoL}}$ is the end-of-life factor, and $\sum_{i=1}^{n}(\boldsymbol{w_i} \times \boldsymbol{BCC_i} \times \boldsymbol{F_{syn,i}})$ is the sum of biobased carbon content multiplied by the respective synthetic factors ($F_{syn}$) and the weight fraction ($\boldsymbol{w_i}$) of each component (see ref. 37). The biobased carbon content (BCC) for PPH and HEMA was determined from Eq. S2 (see ref. 37). Considering the potential production of the precursors of PPH and HEMA, all carbons from their structure were considered biobased in this work (see refs. 31 to 34).

$$\boldsymbol{BCC}(\%) = \left[\frac{C_{renewable}}{C_{renewable}+C_{non-renewable}}\right] \tag{S2}$$

The $F_{syn}$ was determined according to Eq. S3 for each resin component:

$$\boldsymbol{F_{syn}} = \boldsymbol{f_{haz}} \times \boldsymbol{f_{sol}} \times \boldsymbol{f_{T+t}} \times \boldsymbol{AE} \tag{S3}$$

where $\boldsymbol{f_{haz}}$ is related to hazardous chemicals in the synthesis of the respective monomer, $\boldsymbol{f_{sol}}$ is a factor attributed to the sustainability of the solvent used, $\boldsymbol{f_{T+t}}$ is a temperature-time parameter of the synthesis, and AE is the atom economy defined by Eq. S4 (see ref. 37):

$$\boldsymbol{AE} = \frac{\boldsymbol{MW_i}}{\sum_j(\boldsymbol{n_j} \times \boldsymbol{MW_j})} \tag{S4}$$

where $\boldsymbol{MW_i}$ is the molecular weight of component $\boldsymbol{i}$ and $\sum_j(\boldsymbol{n_j} \times \boldsymbol{MW_j})$ is the sum of the molecular weight multiplied by the respective number of equivalents of all reagents $\boldsymbol{j}$ used in the synthesis of 1 equivalent of component $\boldsymbol{i}$. It should be noted that the MW of PPH was assumed to correspond to the oligomeric unit shown in Figure S2.

**Table S1** Biobased carbon content and synthetic factors of each resin component used for the calculation of the Sustainable Formulation Score (see ref. 37).

| Component | $BBC_i$ / % | $f_{haz}$ | $f_{sol}$ | $f_T$ | $f_t$ | $f_{T+t}$ | AE | $F_{syn}$ | $F_{EoL}$ |
|---|---|---|---|---|---|---|---|---|---|
| PPH | 100 | 0.70 | 1.2 | 0.8 | 1.2 | 0.83 | 0.73 | 0.51 | 0.8 |
| HEMA | 100 | 0.70 | 1.2 | 0.8 | 1.0 | 0.80 | 0.88 | 0.59 | 0.8 |

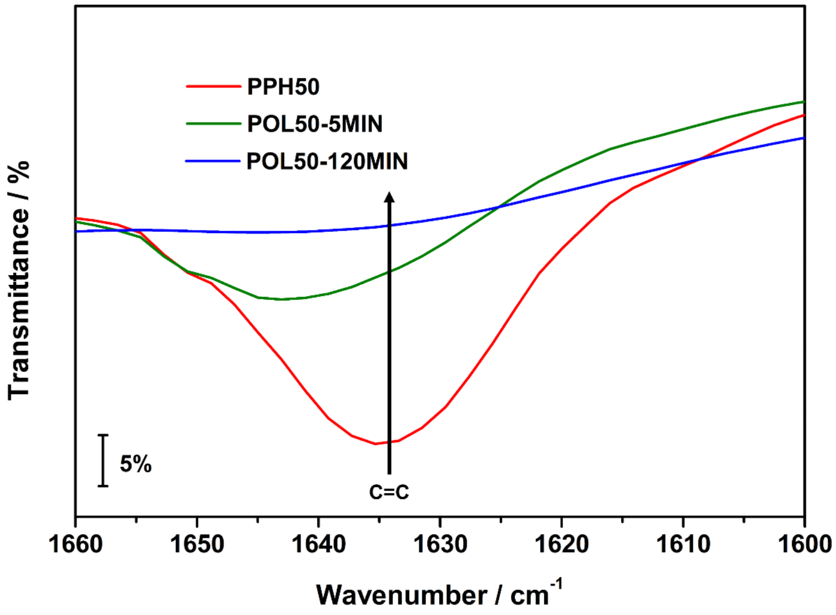


**Figure S3.** MIR spectra of PPH50 and the 3D-printed polymer after 5 and 120 minutes of post-cure under UV (365 nm).

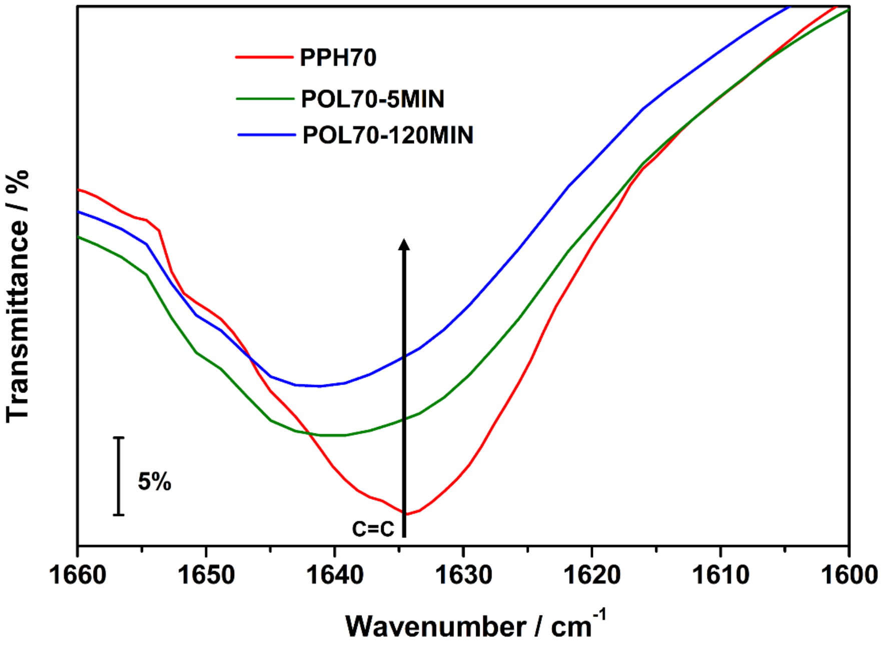


**Figure S4.** MIR spectra of PPH70 and the 3D-printed polymer after 5 and 120 minutes of post-cure under UV (365 nm).

**Table S2.** Temperature ranges (*θ/ ºC*), mass loss values (*Δm/ %*), peak temperatures ($T_p$/ *ºC*), temperature of maximum degradation rate ($T_{MDR}$/ *ºC*), and maximum degradation rate (*MDR/ % min*$^{-1}$) associated with each mass loss stage of the tunned polymers.

| Step | Sample | POL30-120 | POL50-120 | POL70-120 |
|---|---|---|---|---|
| 1[a] | *θ/ °C* | 112.8-250.7 | 134.7-271.9 | 127.5-273.8 |
| | *Δm / %* | 7.5 | 11.0 | 11.9 |
| | $T_p$/ °C | --- | --- | 188.4↑ |
| | $T_{MDR}$/ ºC | --- | 223.3 | --- |
| | *MDR/ % min*$^{-1}$ | --- | 1.2 | --- |
| 2[a] | *θ/ °C* | 250.7-466.1 | 271.9-472.0 | 273.8-459.5 |
| | *Δm / %* | 82.5 | 76.3 | 71.9 |
| | $T_p$/ °C | 377.5↑;431.4↑ | 370.2↑;430.7↑ | 367.9↑;421.8↑ |
| | $T_{MDR}$/ ºC | 255.8;413.9 | 412.9 | 406.6 |
| | *MDR/ % min*$^{-1}$ | 1.3;13.7 | 13.6 | 15.8 |
| 3[a] | *θ/ °C* | 466.1-609.7 | 472.0-621.4 | 459.5-631.1 |
| | *Δm / %* | 9.1 | 12.6 | 16.1 |
| | $T_p$/ °C | 528.4↑ | 534.5↑ | 548.9↑ |
| | $T_{MDR}$/ ºC | 532.8 | 534.1 | 549.9 |
| | *MDR/ % min*$^{-1}$ | 1.4 | 2.1 | 2.1 |

↑ = Exothermic peak.

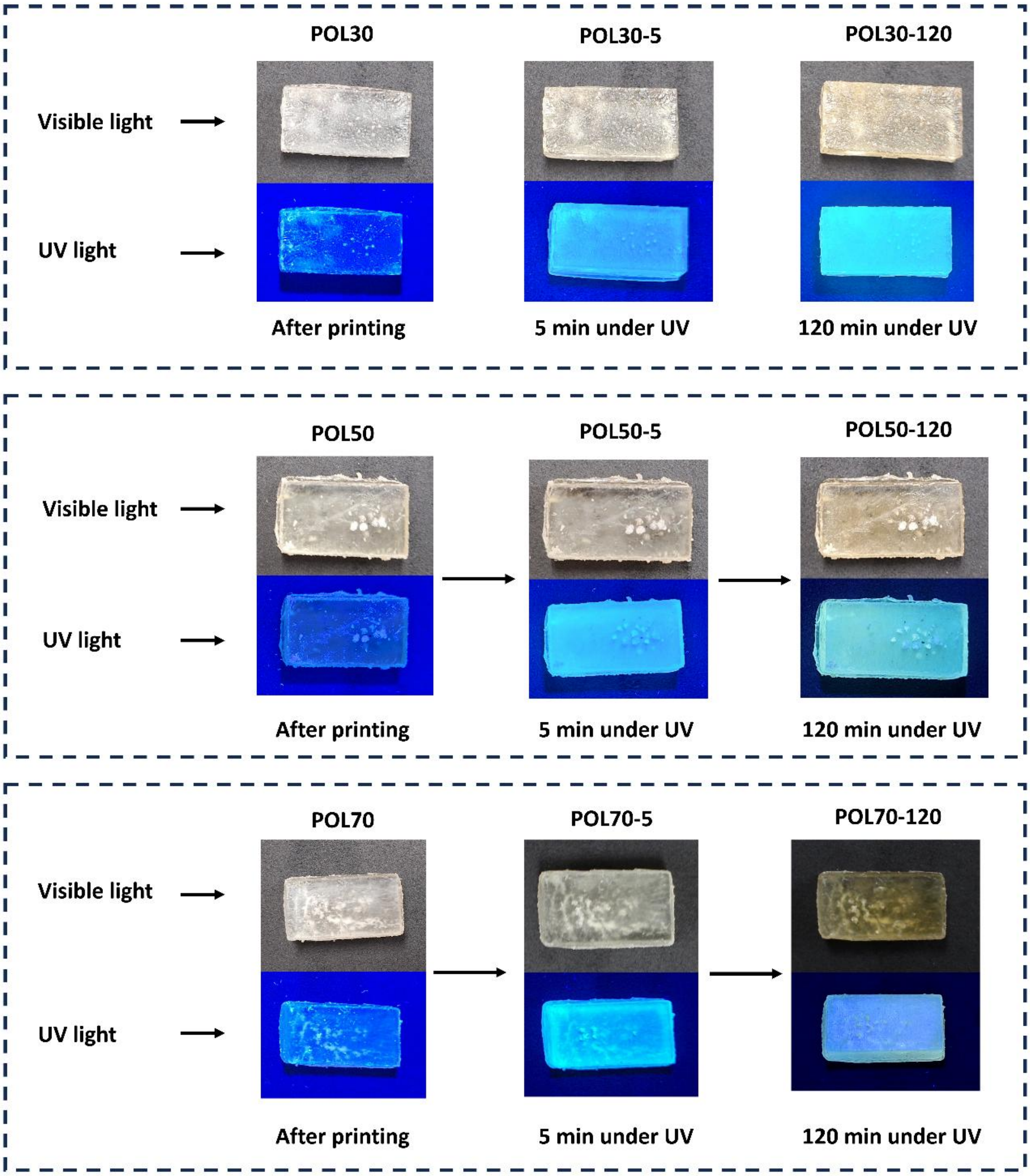


**Figure S5.** Fluorescent behavior of 3D-printed polymers from each resin before and after post-cure under UV light (365 nm) at different times.

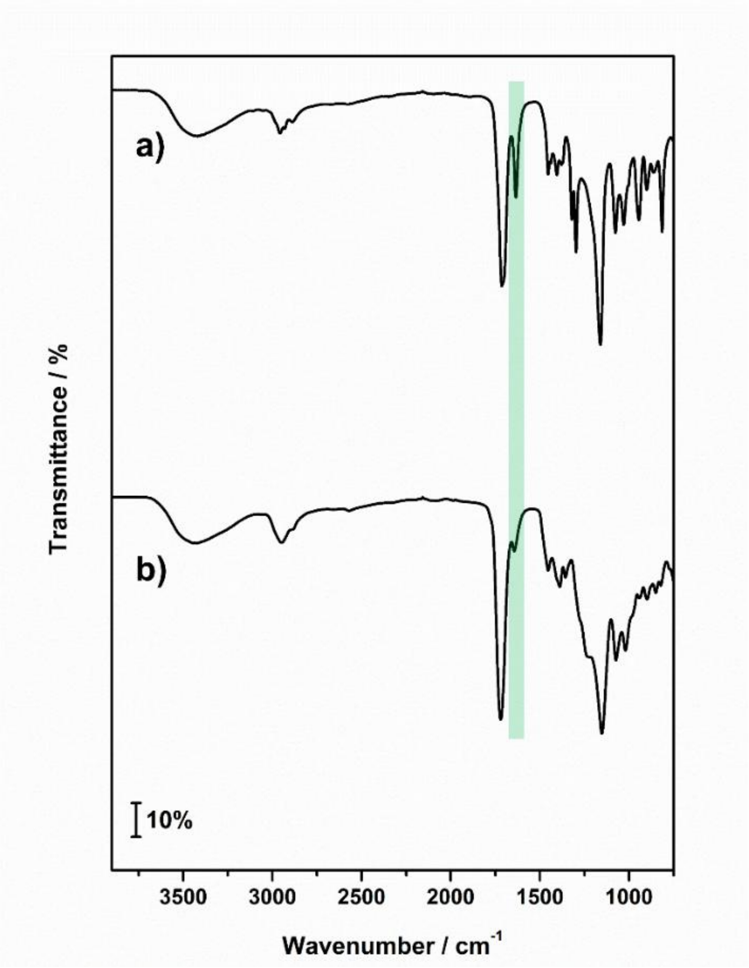


**Figure S6.** MIR spectra for (a) monomeric mixture containing PPH30 and PT3M and (b) polymer produced from this mixture after 5 minutes under UV (365 nm) irradiation.

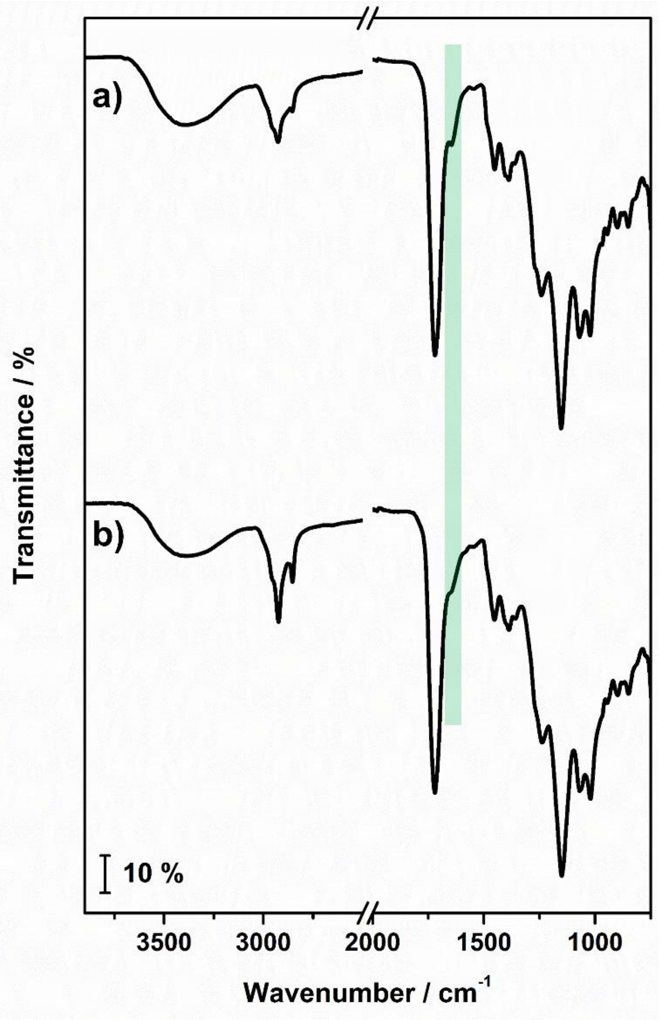


**Figure S7.** MIR spectra for POL30-120 (a) before and (b) after the shape memory test by DMA.
